# Successful Confirmation of Dual Genital Herpes Co-Infection with Herpes Simplex Virus 1 and Herpes Simplex Virus 2 Using Unbiased Metagenomic Next-Generation Sequencing

**DOI:** 10.3390/v15091957

**Published:** 2023-09-20

**Authors:** Chun Kiat Lee, Sau Yoke Ng, Chean Nee Chai, Yu Feng Lim, Tiffany Jingyan Hu, Ogestelli Fabia Lee, Gabriel Yan

**Affiliations:** 1 Department of Laboratory Medicine, National University Health System, Singapore 119228, Singapore; sau_yoke_ng@nuhs.edu.sg (S.Y.N.); chean_nee_chai@nuhs.edu.sg (C.N.C.); yu_feng_lim@nuhs.edu.sg (Y.F.L.); tiffany_jy_hu@nuhs.edu.sg (T.J.H.); gabriel_zherong_yan@nuhs.edu.sg (G.Y.); 2 School of Life and Physical Sciences, PSB Academy, Singapore 039594, Singapore; ogestellifabialee@gmail.com; 3 Department of Medicine, National University Health System, Singapore 119228, Singapore

**Keywords:** HSV, genital herpes, co-infection, metagenomic, next-generation sequencing

## Abstract

Dual co-infection with both HSV-1 and HSV-2 is rare, with few cases reported in the literature. In this case report, we describe the successful use of unbiased metagenomic next-generation sequencing (mNGS) as a rapid and alternative method for confirming dual genital herpes co-infection. Our case involves a 74-year-old woman who presented with genital lesions and initially tested positive for both HSV-1 and HSV-2 via the Luminex ARIES HSV 1&2 assay. The entire mNGS process, from nucleic acid extraction to result analysis, was completed in less than 48 h. Using mNGS, we identified mapped reads specific to either HSV-1 or HSV-2 and screened the sequences to rule out mis-genotyping by the Luminex ARIES assay. Notably, the generated sequences can reveal sequence variations within multiple gene regions, demonstrating the potential of mNGS for identifying novel HSV-1 and HSV-2 variants. Our findings suggest that mNGS can serve as a rapid and reliable alternative confirmatory method for dual genital herpes infections, providing valuable information to guide appropriate treatment options for patients. By eliminating the need for prior knowledge of causative agents, mNGS offers an unbiased approach for detecting and characterizing viral co-infections.

## 1. Introduction

Herpes simplex virus types 1 and 2 (HSV-1 and HSV-2; Family *Herpesviridae*, genus *Simplexvirus*) are double-stranded DNA viruses that can cause meningitis, encephalitis, genital and oral herpes [1,2]. Co-infection with both HSV-1 and HSV-2 is rare, as typically only one type of virus is involved. The accurate detection of HSV co-infection is crucial for guiding appropriate treatment options [1,2,3]. Molecular testing by polymerase chain reaction (PCR) is the most sensitive method of direct detection and differentiation for HSV-1 and HSV-2 [4].

However, confirming co-infection with HSV-1 and HSV-2 can be diagnostically challenging for clinical laboratories, especially when the targeted gene region information of the PCR assay is proprietary or when the clinical laboratory is unable to retest the sample with another reference assay.

In this report, we describe a case of genital herpes co-infection with both HSV-1 and HSV-2, detected with an FDA-approved PCR assay that uses melting curve analysis for HSV detection and differentiation. To ensure the accuracy of the test result, we turned to unbiased metagenomic next-generation sequencing (mNGS) as an alternative method for confirmation. Unbiased mNGS provides an unbiased approach for detecting and characterizing viral co-infections and is well-suited as a complementary tool for confirming dual infections of HSV-1 and HSV-2 [5]. Our results revealed the presence of both HSV-1 and HSV-2 genetic sequences in the patient’s sample, confirming the co-infection.

## 2. Materials and Methods

On 5 April 2021, a swab was collected from a genital lesion of a 74-year-old Chinese woman and placed in universal transport medium (UTM). The sample was sent to the Molecular Diagnosis Centre at the National University Health System in Singapore for HSV PCR testing. The detection of HSV was performed using the ARIES HSV 1&2 assay, an FDA-approved test kit manufactured by Luminex (Luminex Corp., Austin, TX, USA), following the manufacturer’s protocol [6]. Briefly, 200 µL of the UTM sample was transferred into the ARIES cassette chamber and loaded onto the ARIES system (Luminex Corp.) for automated total nucleic acid extraction (TNA), PCR amplification, melting curve analysis, and result interpretation.

The residual UTM sample underwent further laboratory investigation using unbiased mNGS, following previously published method [7]. Briefly, total nucleic acid (TNA) was extracted from 400 µL of UTM using the EZ1 Virus Mini Kit v2.0 (QIAGEN, Hilden, Germany) in a BioRobot EZ1 Advanced XL workstation (QIAGEN), following the manufacturer’s instructions. The elution buffer volume was 60 µL. The extracted TNA was used to generate indexed paired-end libraries with the Nextera XT DNA Library Prep Kit (Illumina Inc., San Diego, CA, USA). The resulting libraries were sequenced on the MiSeq system (Illumina Inc.) using the MiSeq Reagent Kit v2 Micro (2 × 150 reads).

After the sequencing process, we applied a quality control (QC) process which encompassed the removal of low-quality reads and the trimming of adapters using the Trimmomatic tool [8] and host sequence subtraction by mapping the post-QC reads to the hg19 reference genome using BWA version 0.7.17 [9]. The retained reads were then used for our analysis.

The processed reads first underwent a highly sensitive paired-end mapping procedure against the reference genomes of HSV-1 (GenBank accession number: NC_001806.2) and HSV-2 (GenBank accession number: NC_001798.2) using the STAMPY read mapper [10]. STAMPY enables the mapping of reads with substantial divergence from existing reference genomes. This is crucial to prevent the inadvertent exclusion of markedly divergent HSV reads, thereby addressing potential mapping failures.

After STAMPY, the reads associated with “HSV-1” and “HSV-2” were subjected to individual de novo assembly process using the MEGAHIT software [11]. Subsequently, we conducted a BLASTN comparison of the MEGAHIT contigs against the National Center for Biotechnology Information (NCBI) Nucleotide (nr/nt) collection, last updated on 18 August 2023 [12]. Based on the results from the top 10 hits obtained in the BLASTN comparison, we categorized the generated contigs into either HSV-1 or HSV-2 contigs. 

Next, we proceeded to construct partially assembled HSV-1 and HSV-2 genomes by aligning the previously generated contigs to their respective reference genomes. Subsequently, paired-end remapping of the partially assembled genomes was performed using the STAMPY “HSV-1” and “HSV-2” mapped reads, using BWA. To ensure specificity in remapping, we intentionally disabled the realignment option with the Smith-Waterman algorithm for unmapped reads. Lastly, genome coverage plots were generated for the partially assembled genomes of HSV-1 and HSV-2.

## 3. Results

### 3.1. Luminex ARIES HSV 1&2 Assay

**Figure 1:**
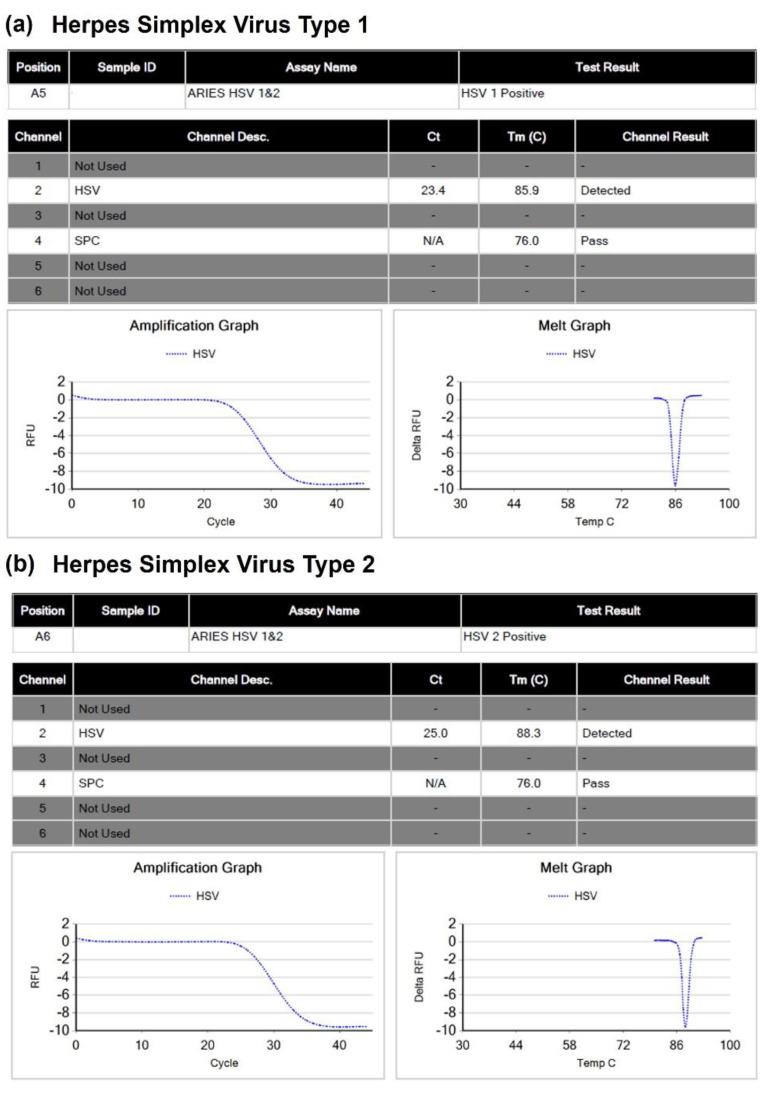

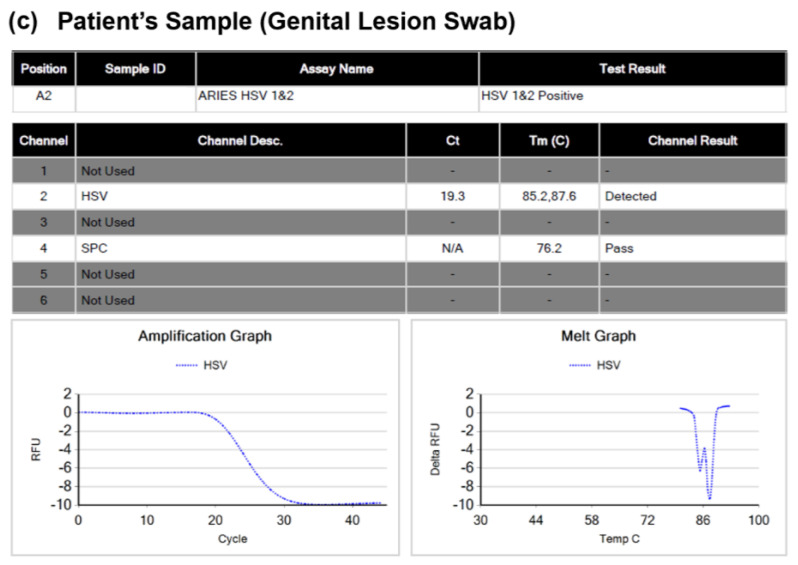
Detailed reports generated by the ARIES system’s onboard instrument software for the ARIES HSV 1&2 assay. (**a**) Herpes simplex virus type 1; (**b**) Herpes simplex virus type 2; (**c**) Report for the patient’s sample (genital lesion swab), which indicates co-infection with both herpes simplex virus type 1 and 2. Note: SPC, Sample processing control.

The ARIES HSV 1&2 assay utilizes a primer pair specific to HSV-1 and HSV-2 for detecting HSV, as well as a second primer pair specific to a sample processing control sequence, which serves as an internal control. Differentiation between HSV-1 and HSV-2 is based on the melting temperature (T_m_) value of the amplified DNA sequences. Figure 1a,b illustrate examples of detailed reports generated by the ARIES HSV 1&2 assay for HSV-1 and HSV-2, respectively. The melting temperature (T_m_) value of HSV-1 ranges from 85.0 to 86.5, while that of HSV-2 ranges from 87.0 to 88.5. Figure 1c displays the detailed report of the patient’s sample, which revealed the presence of both HSV-1 and HSV-2 in the lesion swab sample. The onboard software of the ARIES system detected two distinct T_m_ values, with T_m_ values of 85.2 and 87.6 corresponding to HSV-1 and HSV-2, respectively, in the patient’s sample.

### 3.2. Unbiased Metagenomic-Based Next-Generation Sequencing (mNGS)

A total of 1,686,194 paired-end reads were generated during the sequencing process. After undergoing Trimmomatic-based trimming and low-quality read filtering, 1,641,960 paired-end reads (97.38%) were retained. Following host sequence subtraction, only 11,690 paired-end reads (0.69%) remained for subsequent analysis. After mapping using the STAMPY tool, a total of 433 read pairs (0.03%) were classified as “HSV-1” mapped reads, while 1935 read pairs (0.12%) were classified as “HSV-2” mapped reads. Subsequently, leveraging the MEGAHIT assembly software, 200 contigs were generated from the “HSV-1” mapped reads, while 154 contigs generated from the “HSV-2” mapped reads.

Through BLASTN analysis, 196 contigs with a median contig length of 205.50 bp (interquartile range (IQR): 135.75–277.00 bp) were specific to HSV-1, and 143 contigs with a median contig length of 405 bp (IQR: 221.25–999.25 bp) were specific to HSV-2. This result serves as confirmation of the concurrent presence of HSV-1 and HSV-2 within the patient’s sample. 

After the stringent remapping of the “HSV-1” and “HSV-2” mapped reads to their respective HSV-1 and HSV-2 partially assembled genomes, 384 read pairs (88.68% out of 433) aligned with the HSV-1 genome, whereas 1855 read pairs (95.87% out of 1935) corresponded with the HSV-2 genome.

**Figure 2:**
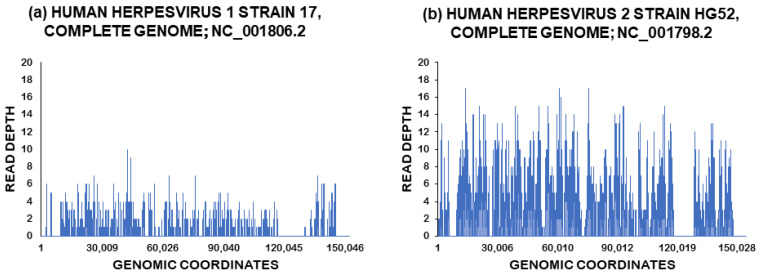
Genome coverage plots of the sequenced herpes simplex virus type 1 and 2 viral genomes in the current study. (**a**) Herpes simplex virus type 1; (**b**) herpes simplex virus type 2. Note: The x-axis displays the genomic coordinates, while the y-axis displays the read depth. The line graph (annotated in blue) displays the genome coverage plot of the virus.

Figure 2a,b display the genome coverage plots of HSV-1 and HSV-2, respectively. In the case of HSV-1, the reads were mapped to various regions within the genome, resulting in a breadth of coverage (reflecting the total percentage of the viral genome covered by sequencing) of 31.18%.

Notably, the genome coverage plot demonstrated a balanced distribution of reads across various genome segments, minimizing the possibility of spurious mapping at a particular position. This factor reinforces our confidence in confirming the detection of HSV-1. Similarly, for HSV-2, the reads were also mapped to different regions within the genome, but the coverage was higher at 63.55%. The depth of sequencing coverage, which indicates the average number of reads for a given nucleotide in the viral genome, was computed at 0.63× for HSV-1 and 3.00× for HSV-2, respectively.

It is likely that many reads mapping to the repeat regions of the HSV genome were discarded because they could match multiple sites on a given genome (this applies to both HSV-1 and HSV-2). Thus, the absence of reads matching in the repeats, as shown in Figure 2, is likely the result of the artifactual removal of these reads during the contig generation stage. This, in turn, would lower the average sequencing coverage across the two genomes.

In a prior investigation conducted by Lamers et al. [13], it was highlighted that the glycoprotein genes of HSV-1 and HSV-2 shared a significant similarity in terms of length and successful alignment. However, an exception arose in the context of *glycoprotein G* (*US4*) gene, where notable differences in gene length between HSV-1 and HSV-2 posed alignment challenges, except for the 3′ ends of the coding sequences. 

In our present study, we executed a manual inspection of the *US4* gene subsequent to the remapping of the HSV-1 and HSV-2 partially assembled genomes using Artemis version 18.2.0 [14]. For HSV-1, an HSV-1-specific contig spanning 193 bp was identified, covering the *US4* gene from nucleotide position 136,823 to 137,015, using GenBank accession number NC_001806.2 as reference. For HSV-2, two distinct contigs of 940 bp and 153 bp were identified. These contigs extended from positions 137,137 to 138,076 and from 138,694 to 138,846, respectively, with reference to GenBank accession number NC_001798.2.

## 4. Discussion

To our knowledge, there have been only two previously reported cases of dual genital herpes co-infection involving both HSV-1 and HSV-2. The first documented case, as reported by Perkins et al., involved a 23-year-old pregnant woman [15]. In their study, confirmation of HSV-1 and HSV-2 co-infection was achieved through a PCR assay, followed by direct immunofluorescent analysis (DFA) using serotype-specific antibodies. The PCR assay yielded results in less than 5 h, while the presence of HSV-2 was detected by DFA at 24 h post-inoculation, followed by the detection of HSV-1 at 48 h post-inoculation. The second reported case was presented by Casto et al., where dual genital herpes co-infection was identified in six individuals [16]. In their study, confirmation of HSV-1 and HSV-2 co-infection was conducted using a TaqMan-based real-time genotyping assay specifically targeting the *UL27* gene of the HSV. Our case represents the third reported instance of such co-infection.

Three other reports of HSV-1 and HSV-2 co-infection have been found in different clinical settings, with the first reporting the detection of herpes co-infection in a rectal swab from a 24-year-old homosexual man with simultaneous proctitis and meningitis [17], the second involving the detection of herpes co-infection in the cerebrospinal fluid from a 62-year-old female patient with seizures and encephalitis [18], and the third report being a case of disseminated neonatal herpes that resulted in the death of a full-term infant [19].

For a patient with seizures and encephalitis, Anderson et al. confirmed the dual infection using two separate PCR assays. The first PCR used melting curve analysis for differentiation between HSV-1 and HSV-2, while the second PCR utilized an FDA-cleared assay with fluorescent probes for specific detection and differentiation between the two HSV types [18]. 

In the fatal case of disseminated neonatal herpes, PCR was performed on all postmortem samples and virus-positive cultures, followed by Sanger sequencing for confirmation. Retrospective serological examination of maternal samples and avidity tests were also conducted, which detected the presence of IgM and IgG antibodies for both HSV types. The increase in IgG avidity suggested primary infection [19].

Despite the limited number of published cases documenting co-infections of HSV-1 and HSV-2, it is worth noting that seroprevalence studies in the United States have revealed a relatively high rate of co-infections, with more than 10% of individuals testing positive for both HSV-1 and HSV-2 antibodies [20,21]. However, it is important to understand that these prevalence figures are derived from blood samples which were tested to detect the presence of antibodies specific to HSV-1 and HSV-2. This implies that while clinical cases of co-infection might be relatively rare or underreported, a significant portion of the population may have been exposed to both types of herpes viruses, although not necessarily at the same time.

Determining whether co-infection is truly present can be challenging for clinical laboratories, especially when the targeted gene region information of the molecular detection assay is proprietary or when the laboratory cannot perform orthogonal confirmation due to the unavailability of a reference assay. In this study, we demonstrate the clinical utility of unbiased mNGS as a rapid and alternative method for confirming dual genital herpes co-infection with both HSV-1 and HSV-2, which was initially detected by the ARIES HSV 1&2 assay. The PCR assay yielded results within 2 h, while the mNGS process, from TNA extraction to sequencing result analysis, took less than 48 h.

From the generated sequencing data, we identified mapped reads specific to either HSV-1 or HSV-2 and screened the read sequences to rule out the presence of sequence variants that may have resulted in mis-genotyping by the ARIES HSV 1&2 assay. Our results confirmed the co-infection by revealing the presence of both HSV-1 and HSV-2 in the patient’s sample. Overall, the viral titer of HSV-2 appears to be higher than that of HSV-1, as indicated by the number of distinct read pairs that are specific to HSV-1 (n = 384) and HSV-2 (n = 1855). This finding is consistent with the melting curve analysis of the ARIES HSV 1&2 assay, which shows a higher delta relative fluorescence unit for HSV-2 compared to HSV-1 (see Figure 1c). However, it is important to note that the ARIES HSV 1&2 assay is intended for the qualitative detection of HSV. 

The *glycoprotein G* (*US4*) gene has been previously reported to exhibit significant differences in gene length between HSV-1 and HSV-2 [13]. Consequently, this divergence has led to the historical utilization of the protein product of glycoprotein G to discern between HSV-1 and HSV-2 antibody responses. Leveraging this characteristic, we conducted a manual examination of the mapped contigs encompassing the *US4* gene region to further confirm the co-presence of both HSV-1 and HSV-2 within the patient’s sample. As a result of this in-depth analysis, we successfully identified one HSV-1-specific contig and two HSV-2-specific contigs, providing additional evidence to support the co-infection scenario.

In conclusion, our study showcases the successful application of unbiased mNGS as a confirmatory method for dual genital herpes co-infection with both HSV-1 and HSV-2. This approach does not require prior knowledge of the causative agents for detection [5]. Additionally, the generated sequences can be used to identify variations within multiple gene regions simultaneously to detect novel HSV-1 and HSV-2 variants, which may result in mis-genotyping by assays that rely on a specific gene region. Therefore, mNGS provides a promising tool for improving patient care, serving as an alternative confirmatory method for dual infections.

## Data Availability

The data presented in this study are available on request from the corresponding author. The data are not publicly available due to privacy and ethical concerns.
